# A review of harmonization strategies for quantitative PET

**DOI:** 10.1007/s12149-022-01820-x

**Published:** 2023-01-06

**Authors:** Go Akamatsu, Yuji Tsutsui, Hiromitsu Daisaki, Katsuhiko Mitsumoto, Shingo Baba, Masayuki Sasaki

**Affiliations:** 1 Department of Advanced Nuclear Medicine Sciences, Institute for Quantum Medical Sciences, National Institutes for Quantum Science and Technology (QST), 4-9-1 Anagawa, Inage-ku, Chiba, 263-8555 Japan; 2 Department of Molecular Imaging Research, Kobe City Medical Center General Hospital, 2-1-1 Minatojima-minamimachi, Chuo-ku, Kobe, Hyogo 650-0047 Japan; 3 Department of Radiological Science, Faculty of Health Science, Junshin Gakuen University, 1-1-1 Chikushigaoka, Minami-ku, Fukuoka, 815-8510 Japan; 4 Department of Radiological Technology, Gunma Prefectural College of Health Sciences, 323-1 Kamioki-machi, Maebashi, Gunma 371-0052 Japan; 5 Department of Clinical Radiology Service, Kyoto University Hospital, 54 Shogoin-Kawaharacho, Sakyo-ku, Kyoto, 606-8507 Japan; 6 Department of Clinical Radiology, Graduate School of Medical Sciences, Kyushu University, 3-1-1, Maidashi, Higashi-ku, Fukuoka, 812-8582 Japan; 7 Department of Medical Quantum Science, Faculty of Medical Sciences, Kyushu University, 3-1-1, Maidashi, Higashi-ku, Fukuoka, 812-8582 Japan

**Keywords:** PET, Harmonization, Standardization, SUV, Quantification

## Abstract

PET can reveal in vivo biological processes at the molecular level. PET-derived quantitative values have been used as a surrogate marker for clinical decision-making in numerous clinical studies and trials. However, quantitative values in PET are variable depending on technical, biological, and physical factors. The variability may have a significant impact on a study outcome. Appropriate scanner calibration and quality control, standardization of imaging protocols, and any necessary harmonization strategies are essential to make use of PET as a biomarker with low bias and variability. This review summarizes benefits, limitations, and remaining challenges for harmonization of quantitative PET, including whole-body PET in oncology, brain PET in neurology, PET/MR, and non-^18^F PET imaging. This review is expected to facilitate harmonization of quantitative PET and to promote the contribution of PET-derived biomarkers to research and development in medicine.

## Introduction

Positron emission tomography (PET) can measure in vivo biological processes at the molecular level. In clinical practice, PET is an essential imaging modality for diagnosis of various diseases [1]. Furthermore, PET can be used as a research tool to elucidate human physiological and pathological processes. The advantage of PET imaging is its high quantitative accuracy [2]. Various quantitative metrics are used according to the purpose of each PET imaging. The most popular metric is a standardized uptake value (SUV), which is normalized by body weight. The SUV is calculated by the following equation:

$$\mathrm{SUV}= \frac{{\mathrm{AC}}_{\mathrm{VOI}} (\mathrm{kBq}/\mathrm{mL})}{\mathrm{ID} \left(\mathrm{MBq}\right) / \mathrm{BW} (\mathrm{kg})},$$

where *AC*_VOI_ is the average (or the maximum) activity concentration in the specified volume of interest (VOI), *ID* is the injected dose of radiopharmaceuticals, and *BW* is the body weight. The SUV is a unitless metric based on the assumption that human tissue density is equal to the density of water (1 g = 1 mL). Tracer uptakes in specified regions can be quantitatively evaluated with SUVs.

In treatment response assessment studies, quantitative metrics such as SUVs and their percentage change can be a surrogate marker to assess the therapeutic response [3–5]. For assessing the therapeutic response using pre-therapy and follow-up FDG-PET, changes in tumor SUVs have been used as primary and secondary endpoints in numerous studies and trials [4, 6, 7]. In EORTC criteria and PERCIST [8, 9], the tumor response is classified into four categories: complete metabolic response (CMR), partial metabolic response (PMR), stable metabolic disease (SMD), and progressive metabolic disease (PMD). In such cases, SUVs would be one of the key biomarkers for clinical decision-making.

**Fig. 1:**
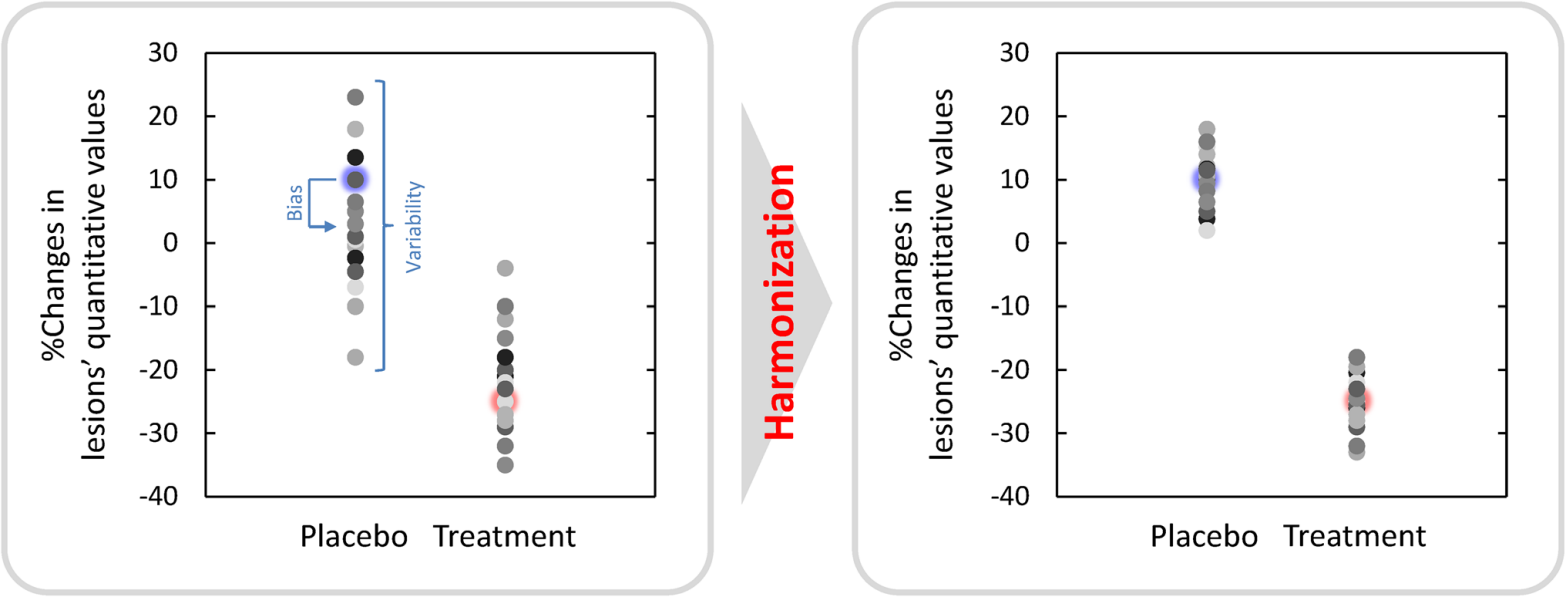
Expected merits of harmonization of quantitative values in PET. True changes were + 10% (the blue point) for the placebo group and − 25% (the red point) for the treatment group. Bias and variability in quantitative values are reduced by harmonization

However, quantitative values derived from PET are variable depending on technical, biological, and physical factors [10–12]. In multicenter studies using quantitative metrics, the variability of the metrics may have a significant impact on the study outcomes [13, 14]. Table 1 summarizes main factors affecting SUVs. Appropriate scanner calibration and quality control effectively reduce technical errors. Standardization of imaging protocols including patient preparation can reduce the unwanted biological bias and variability. Finally, any necessary harmonization strategies should be implemented to minimize the inter-scanner physical variability. These three-steps are key to get reliable (repeatable) and comparable (reproducible) quantitative values. Figure 1 presents the expected merits of harmonization. Harmonization leads to precise outcomes even with small datasets, because the non-pathophysiological bias and variation can be removed [15]. To appropriately use PET as an imaging biomarker, the benefits, limitations, and remaining challenges for harmonization of PET should be known. This review summarizes harmonization strategies for quantitative PET, including whole-body PET in oncology, brain PET in neurology, PET/MR, and non-^18^F emerging applications.

**Table 1:** Summary of factors affecting the SUV

Category	Main factors
Technical factors	Quality control and quality assurance for a PET system and other instruments
	Cross calibration between a PET system and dose calibrator
	Residual radioactivity in syringes and tubes
	Synchronization of clocks between a PET system and dose calibrator
	Decay correction of injected radioactivity
Biological factors	Uptake duration
	Patient motion and breathing
	Patient preparation (fasting, resting condition, etc.)
	Blood glucose level (FDG)
	Inflammation (FDG)
Physical factors	Acquisition duration
	Image reconstruction parameters
	ROI definition
	Normalization factors for SUV (body weight, lean body mass, etc.)
	Attenuation correction position mismatch

### Harmonization strategies for whole-body PET in oncology

**Fig. 2:**
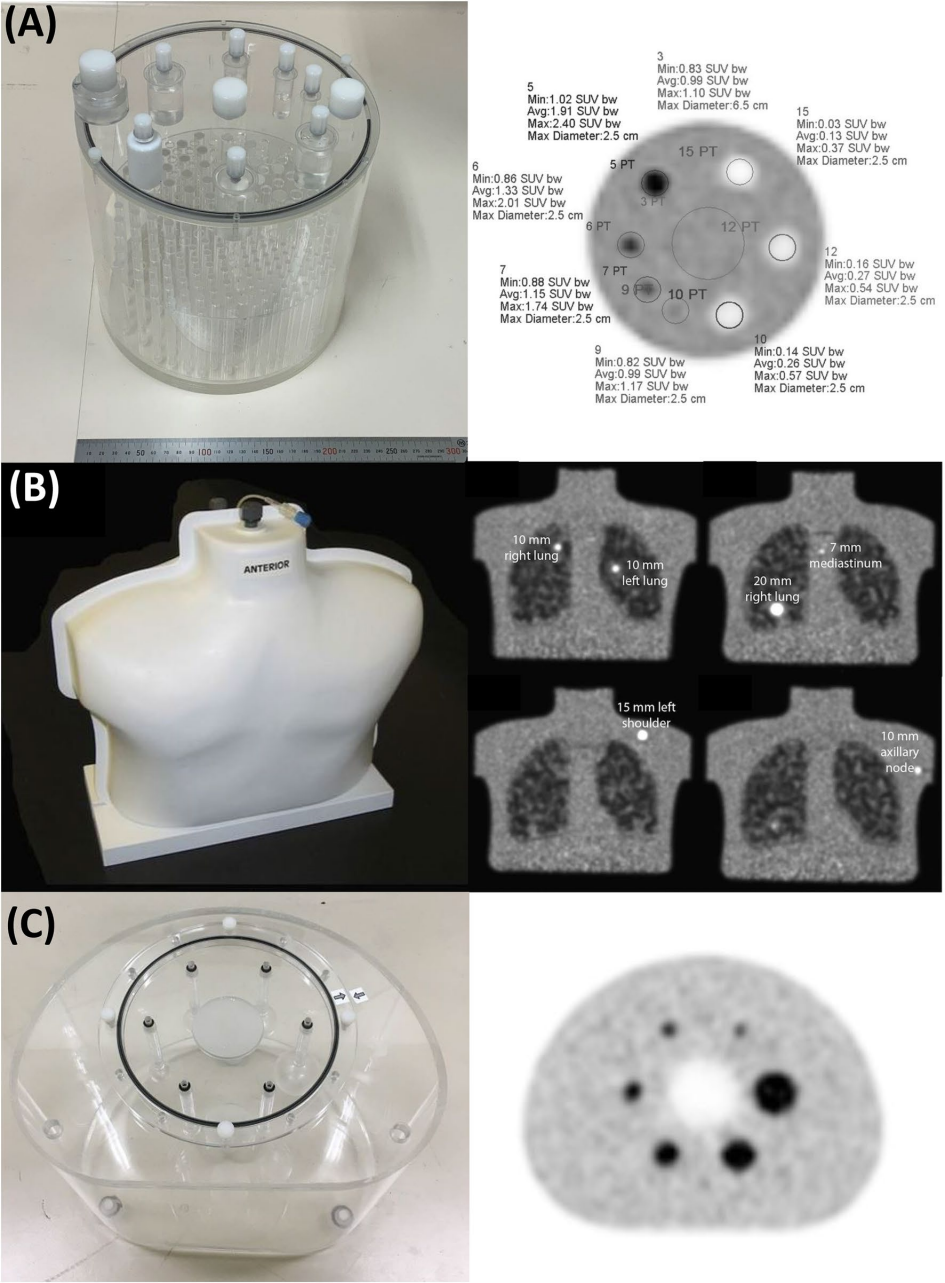
Photographs and PET images of representative phantoms used for harmonization: ACR phantom and its PET image (**A**), SNMMI-CTN chest phantom and its PET images (**B**), and NEMA NU-2 image quality phantom and its PET image (**C**). The PET image of the ACR phantom is reprinted from the paper by DiFilippo et al. [34]. The photograph and PET images of the SNMMI-CTN chest phantom are reprinted from the paper by Sunderland et al. [36]. These studies were originally published in *JNMT* and *JNM*, respectively. © SNMMI

Several organizations have constructed scanner qualification programs for whole-body FDG-PET imaging in oncology. All programs involve standardization of imaging protocols and image quality, and some programs focus more on harmonization of quantitative metrics based on phantom experiments. Figure 2 shows representative phantoms used for harmonization of PET and Table 2 summarizes studies related to PET harmonization [13, 14, 16–34]. While earlier studies focused only on the variability in SUVs, recent studies have assessed clinical outcomes for various cancers using various quantitative metrics such as textural features. PET harmonization can drive the use of PET in clinical oncology studies.

**Table 2:** Publications relating to harmonization of PET

Publications	Phantoms and materials	Metrics and tools	Harmonizing parameters	Evaluation items and outputs
Makris et al. (2013) [16]	NEMA NU2 IQ phantom, ACR phantom, CTN phantom	VOIA50%, VOImax, VOI3Dpeak, VOI2Dpeak	Data acquisition, processing, analysis	Variability
Quak et al. (2015) [17]	NEMA NU2 IQ phantom	Software tool for harmonization	Scanners, reconstruction methods	Variability
Quak et al. (2016) [13]	NEMA NU2 IQ phantom	Software tool for harmonization	Scanners, reconstruction methods	PERCIST classification
Lasnon et al. (2016) [18]	NEMA NU2 IQ phantom	Recovery coefficient	Reconstruction methods	Textural features
Lasnon et al. (2017) [19]	NEMA NU2 IQ phantom	Recovery coefficient	Scanners, reconstruction methods	PERCIST and EORTC classifications
Panetta et al. (2017) [20]	NEMA NU2 IQ phantom	Contrast recovery coefficient	Scanners, reconstruction methods	Agree of CRC
Tsutsui et al. (2018) [21]	NEMA NU2 IQ phantom	Software tool for harmonization	Scanners, reconstruction methods	Repeatability, reproducibility
Kaalep et al. (2018) [22]	NEMA NU2 IQ phantom	Recovery coefficient, SUVmax, SUVmean, SUVpeak	Scanners, reconstruction methods	Variability
Orlhac et al. (2018) [23]	None	ComBat	Scanners, reconstruction methods	Radiomic feature, SUV
Namías et al. (2018) [24]	Cylindrical phantom	Contrast recovery coefficient (simulation)	Scanners, reconstruction methods	Variability
Rubello et al. (2018) [25]	NEMA NU2 IQ phantom, Jaszczak phantom	Recovery coefficient, software tool for harmonization	Scanners, reconstruction methods	Variability
Kaneta et al. (2018) [26]	NEMA NU2 IQ phantom	SUVmax, SUVpeak, software tool for harmonization	Scanners, reconstruction methods	Variability
Kaalep et al. (2019) [27]	NEMA NU2 IQ phantom	SUVmax, SUVmean	Scanners, reconstruction methods	Variability
Houdu et al. (2019) [14]	NEMA NU2 IQ phantom	Software tool for harmonization	Reconstruction methods	Prognosticator
Machado et al. (2019) [28]	NEMA NU2 IQ phantom	Contrast recovery coefficient	Reconstruction methods, scan times, activity	Achieving standards
Daisaki et al. (2021) [29]	NEMA NU2 IQ phantom	SUVmax, software tool for harmonization	Scanners, reconstruction methods	Inter-scanner COVs
Prenosil et al. (2022) [30]	NEMA NU2 IQ phantom	Recovery coefficient	Reconstruction methods, scan times	Variability
Monsef et al. (2022) [31]	NEMA NU2 IQ phantom	SUVmax, SUVmean, SUVpeak	Scanners, reconstruction methods	Variations in SUV measurements
Jiménez–Ortega et al. (2022) [32]	NEMA NU2 IQ phantom	Recovery coefficient, SUVmax, SUVmean, SUVpeak	Reconstruction methods	Treatment planning and monitoring
Akamatsu et al. (2022) [33]	NEMA NU2 IQ phantom	SUVmax, SUVpeak	Scanners, reconstruction methods	Variability

The American College of Radiology Imaging Network (ACRIN) has verified the accuracy of average SUVs using a uniform cylindrical phantom (5.92–8.88 or 8.14 kBq/mL; 6283 or 9293 mL). A 90% circular region-of-interest (ROI) of the interior diameter was applied to the phantom image, and the acceptable average SUV was 1.0 ± 0.1. Scheuermann et al. [35] reported that 12% of scanners (12/101) they tested failed due to incorrect SUV or normalization calibrations. This result suggested that verification of accurate SUV calibration is very important in multicenter studies.

The Society of Nuclear Medicine and Molecular Imaging (SNMMI) Clinical Trial Network (CTN) uses an anthropomorphic chest phantom with fillable spheres for validating the quantitative performance of PET/CT scanners [36, 37]. The standard radioactivity concentration ratio between the spheres and background are 4:1. SUVmax for small spheres and SUVmean for background regions were measured to assess the scanner calibration accuracy and quantitative performance for small lesions [36]. The acceptance criterion for the SUVmean of the uniform background was 1.0 ± 0.1.

**Fig. 3:**
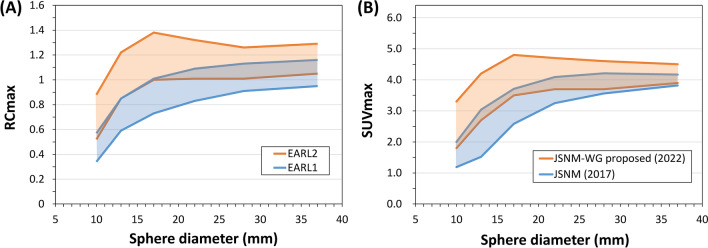
Harmonization ranges for maximum recovery coefficients, which were proposed by EANM (**A**) and JSNM (**B**). The range of the 5th-to-95th percentiles of SUVmax data for 23 scanners is shown here as the JSNM-WG proposed range

In 2010, the European Association of Nuclear Medicine (EANM) Research Ltd. (EANM/EARL) launched a PET/CT scanner accreditation program [38]. The program uses a NEMA NU-2 image quality phantom to measure SUV recovery coefficients in relation to sphere size (diameters: 10–37 mm). The phantom is filled with ^18^F solutions, and the sphere-to-background radioactivity concentration ratio is 10 (20 and 2 kBq/mL) [22]. Maximum, mean, and peak recovery coefficients are measured to check whether they are within pre-specified upper and lower limits. High-resolution scanners sometimes required down-smoothing to fit this range. Consequently, the first standard limits EARL1 were updated to EARL2 according to the progress in PET instrumentation and reconstruction technology (Fig. 3A) [38, 39].

The Radiological Society of North America/Quantitative Imaging Biomarker Alliance (RSNA/QIBA) created the FDG-PET/CT profile to characterize and reduce the variability in SUVs. The QIBA profile comprehensively covers acquisition, reconstruction and post-processing, and analysis and interpretation to obtain SUVmax with a within-subject coefficient of variation (wCV) of 10–12% [40, 41]. Conforming to this profile supports the claim that an increase in SUVmax of 39% or more, or a decrease of 28% or more, indicates that a true biological change has occurred with 95% confidence.

The Japanese Society of Nuclear Medicine (JSNM) has published standard PET imaging protocols and phantom test procedures and criteria to standardize the methods that would affect image quality and quantification accuracy. A NEMA NU-2 image quality phantom is used with a sphere-to-background radioactivity concentration ratio of 4. For SUV harmonization, the SUVmax of hot spheres must satisfy the specified range (Fig. 3B) [33, 42]. A post-smoothing process was sometimes needed for high-resolution systems to meet this range, just as for the EARL limits. In 2022, a JSNM working group proposed new upper and lower ranges for SUVmax recovery curves [33]. This SUV harmonization range was proposed based on NEMA NU-2 image quality phantom data measured with 23 PET/CT scanners. The working group also suggested image quality criteria to ensure the 10 mm sphere visibility and to reduce the intra-scanner variability of quantitative metrics: the contrast-to-noise ratio (Q_H,10 mm_/N_10mm_) should be ≥ 2.5 and the coefficient-of-variance in the background (CV_BG_) should be ≤ 10% (or 14.1%).

Since recent PET systems with advanced reconstruction algorithms may provide higher quantitative values, downgrading image resolution may be needed to harmonize quantitative PET data to those of older PET systems. Such a harmonization process may lose the superior imaging capability of the newer PET systems. One solution to retain the high image quality provided by newer PET systems has been to make a second set of images to provide comparable quantitative values [43]. However, this introduced a cumbersome PET data handling issue, and a better strategy was needed.

To overcome the double dataset handling issue, Kelly et al. [44] developed software that can calculate the harmonized SUVs in the background while displaying PET images reconstructed by an advanced algorithm. It is possible to provide the harmonized SUVs based on a single dataset without losing the advantage of high-resolution PET images. Quak et al. [17] successfully harmonized SUVs of 517 cancer patients using the commercial software, EQ.PET (Siemens). This software can apply the harmonization method of Kelly et al. to clinical data. The harmonization process of the EQ.PET is summarized as follows. First, a maximum recovery coefficient is measured for each sphere in the NEMA NU-2 image quality phantom. These values are then compared to a reference recovery curve to calculate the root mean square error (RMSE). This comparison is repeated while increasing the full width at half maximum (FWHM) of a Gaussian filter. The FWHM value at which the minimum RMSE is obtained then is applied to PET images for harmonization.

**Fig. 4:**
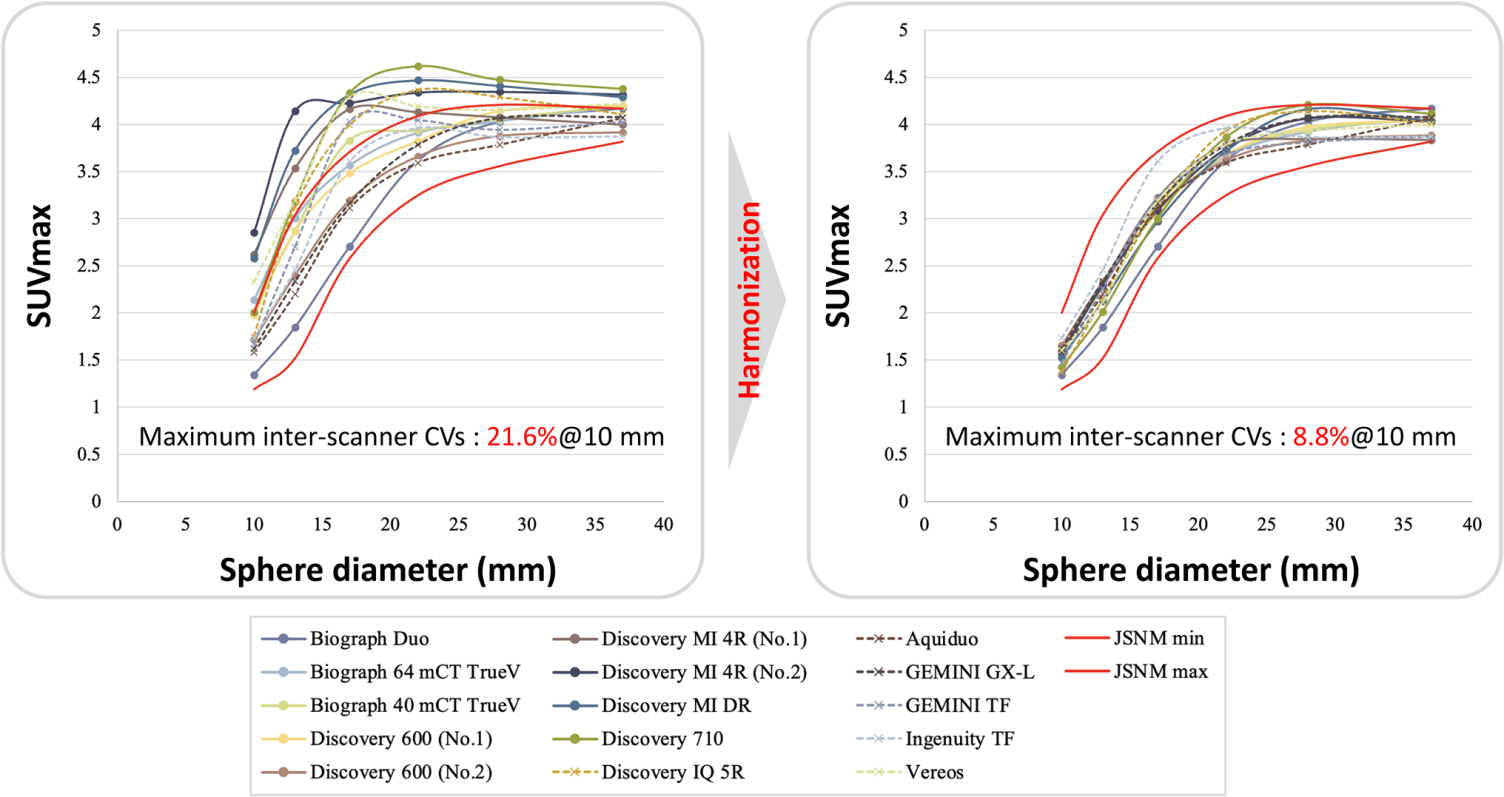
SUVmax recovery curves before and after harmonization using RAVAT. The inter-scanner coefficient-of-variance (CV) is reduced by harmonization

Free vendor-neutral software is desirable for widespread application of this harmonization method. Daisaki et al. [29] successfully harmonized SUVs using the “RC Tool for Harmonization” (Nihon Medi-Physics) which semi-automatically calculates an optimal FWHM of the Gaussian filter and RAVAT (Nihon Medi-Physics) which calculates QIBA Profile-compliant quantitative values. They harmonized SUV recovery curves of 15 PET systems to the JSNM harmonization range (Fig. 4). These vendor-neutral software programs can be freely applied to PET images, irrespective of scanner models. Two datasets are not required, because RAVAT applies a pre-determined additional smoothing filter to PET images. As mentioned below, several clinical studies have used RAVAT software for harmonization of quantitative PET [45, 46].

### Harmonization strategies for brain PET

For brain PET, there are many kinds of radiotracers that can measure metabolisms, protein aggregates, transporters, and receptors [47]. Depending on radiotracer characteristics, various quantitative metrics are used to assess tracer uptakes. For harmonization, brain phantoms are generally used, because image characteristics such as tracer distribution, uptake contrast ratio, and image noise levels for brain PET are different from those for whole-body PET.

**Fig. 5:**
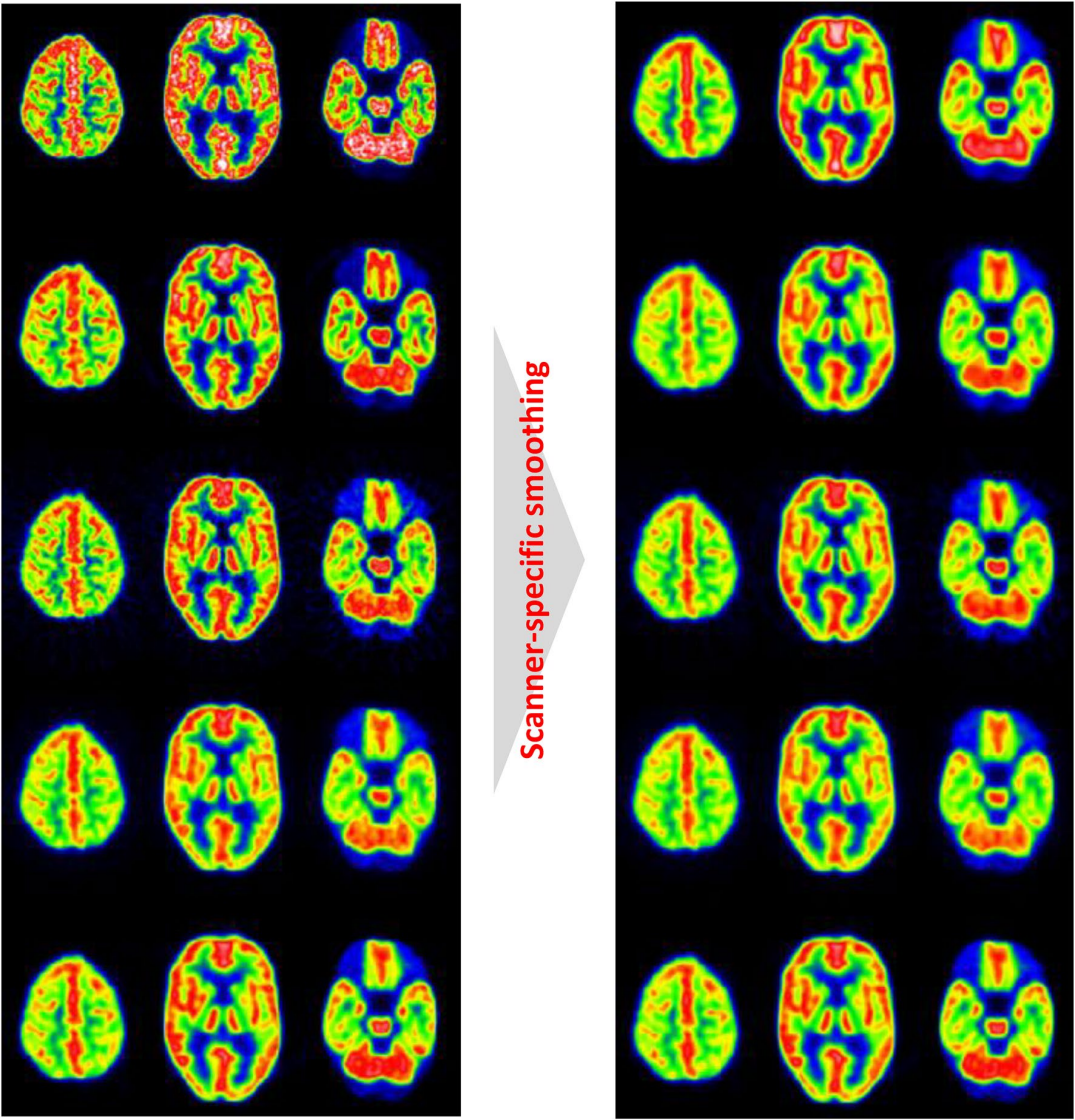
Hoffman phantom images of five PET scanner models with pre- and post-smoothing. After application of the smoothing filter, the visual difference in resolution was reduced. The images are reprinted with modification from the paper by Joshi et al. *Neuroimage*. 2009; 46: 154–159 [48]. Copyright © 2009 Elsevier Inc. All rights reserved

In the multicenter Alzheimer’s Disease Neuroimaging Initiative (ADNI) study, Joshi et al. [48] proposed the two-step harmonization method with the Hoffman 3D brain phantom. Using the digital Hoffman phantom image smoothed with the 8 mm FWHM Gaussian filter as the target resolution, they selected an FWHM of the Gaussian filter for each scanner model. Figure 5 shows representative PET images applied with and without a scanner-specific smoothing filter. Joshi et al. reported that the scanner-specific smoothing approach effectively reduced the inter-scanner variability, while the low frequency correction was not effective. Ikari et al. [49] also used the image resolution of 8 mm FWHM as a reference level. In the Japanese-ADNI (J-ADNI) study, the image resolution was harmonized by a scanner-specific smoothing filter [50]. There are some reports using gray matter contrast recovery (RC_GM_) and gray-to-white matter contrast (GMWMr) of the Hoffman 3D brain phantom [49, 51, 52]. Like the harmonization approach for whole-body PET, Verwer et al. [51] proposed upper and lower limits for RC_GM_ and GMWMr to harmonize image contrast.

Even though the Hoffman 3D brain phantom is widely used, it can only simulate the distribution pattern of FDG in the brain [53]. Other phantoms might be better model for radioactivity distributions other than those of FDG. Hoye et al. [54] harmonized ^11^C-raclopride brain PET images measured by HRRT (high-resolution research tomograph, Siemens) and HR + (a standard clinical scanner, Siemens) using a 3D brain phantom (the Iida phantom [55]). Fahey et al. [56] evaluated image uniformity, spatial resolution, and image quality of 13 PET scanners using the SNMMI CTN brain phantom, which has a uniform section, a resolution section, and a clinical brain simulation section.

### Clinical studies with harmonized PET

Quantitative values in PET can be used as a biomarker in clinical studies once harmonizing of PET data is completed. In oncology fields, PET imaging has been involved in many therapeutic studies [57–62]. Ito et al. [45] assessed anti-PD-1 therapy response to non-small cell lung cancer (NSCLC) using harmonized FDG-PET. They used nine PET/CT scanners and harmonized SUVs based on the JSNM harmonization strategy before applying EORTC criteria and PERCIST. Changes in harmonized SUVs correlated well with overall survival of NSCLC patients. Kitajima et al. [63] investigated relationships of prognosis for stage I–III breast cancer patients and pre-treatment FDG-PET/CT-derived quantitative metrics. They harmonized maximum SUVs of the image quality phantom among five PET/CT scanners according to the JSNM harmonization method. Primary tumor and nodal maximum SUVs and total lesion glycolysis (TLG), which are derived from pre-treatment FDG-PET/CT, were associated with recurrence-free and overall survivals in patients with operable breast cancer.

Among brain PET studies, Sevigny et al. [64] reported that Aducanumab, a human monoclonal antibody that selectively accumulates with Aβ aggregates, reduced Aβ plaques with amyloid PET as an adjunct marker for Aβ pathology. They harmonized amyloid PET images to be a uniform spatial resolution of 6.5 mm in-plane and 7.5 mm axially [65]. Change in amyloid PET SUVR values was used as a surrogate marker for treatment response. Senda et al. [66] performed a multicenter observational study on potential preclinical and prodromal Alzheimer’s disease. Image reconstruction parameters for each PET scanner in the observational study were determined with the Hoffman 3D brain phantom and the uniform cylindrical phantom so that all the scanners met previously established image quality criteria [49]. Senda et al. conducted brain FDG, amyloid and tau PET imaging, and measured AD t-sum values for FDG images and SUVRs for amyloid and tau images.

### Post-reconstruction data-driven harmonization methods for PET

Adjusting reconstruction settings and applying additional smoothing filter are major approaches for PET harmonization. These methods are categorized into “image-based” harmonization approach, because “harmonized images” need to be generated before measuring quantitative metrics in PET images. On the other hand, several data-driven harmonization methods have been applied for quantitative metrics in PET [67].

The centiloid scale (CL) is an example of popularly used data-driven harmonization methods. The CL is a harmonized quantitative metric for amyloid PET proposed by Klunk et al. [68]. The standard CL is calculated from the standardized uptake value ratio (SUVR) of amyloid PET as follows:

$$\mathrm{Centiloid \,scale }\left(\mathrm{CL}\right)=\frac{{\mathrm{SUVR}}_{\mathrm{IND}}-{\mathrm{SUVR}}_{\mathrm{YC}-0}}{{\mathrm{SUVR}}_{\mathrm{AD}-100}-{\mathrm{SUVR}}_{\mathrm{YC}-0}} \times 100,$$

where SUVR_IND_ is an SUVR value of an individual, SUVR_YC-0_ is the mean SUVR of 34 young healthy controls, and SUVR_AD-100_ is the mean SUVR of 45 AD patients. The standard image datasets and the VOI template are available online (https://www.gaain.org). SUVR values measured using various scanners and different amyloid tracers were converted to the unified 0 to 100 scale [69–71]. The CL scale has been applied to a tau PET SUVR conversion by Yamao et al. [72].

**Fig. 6:**
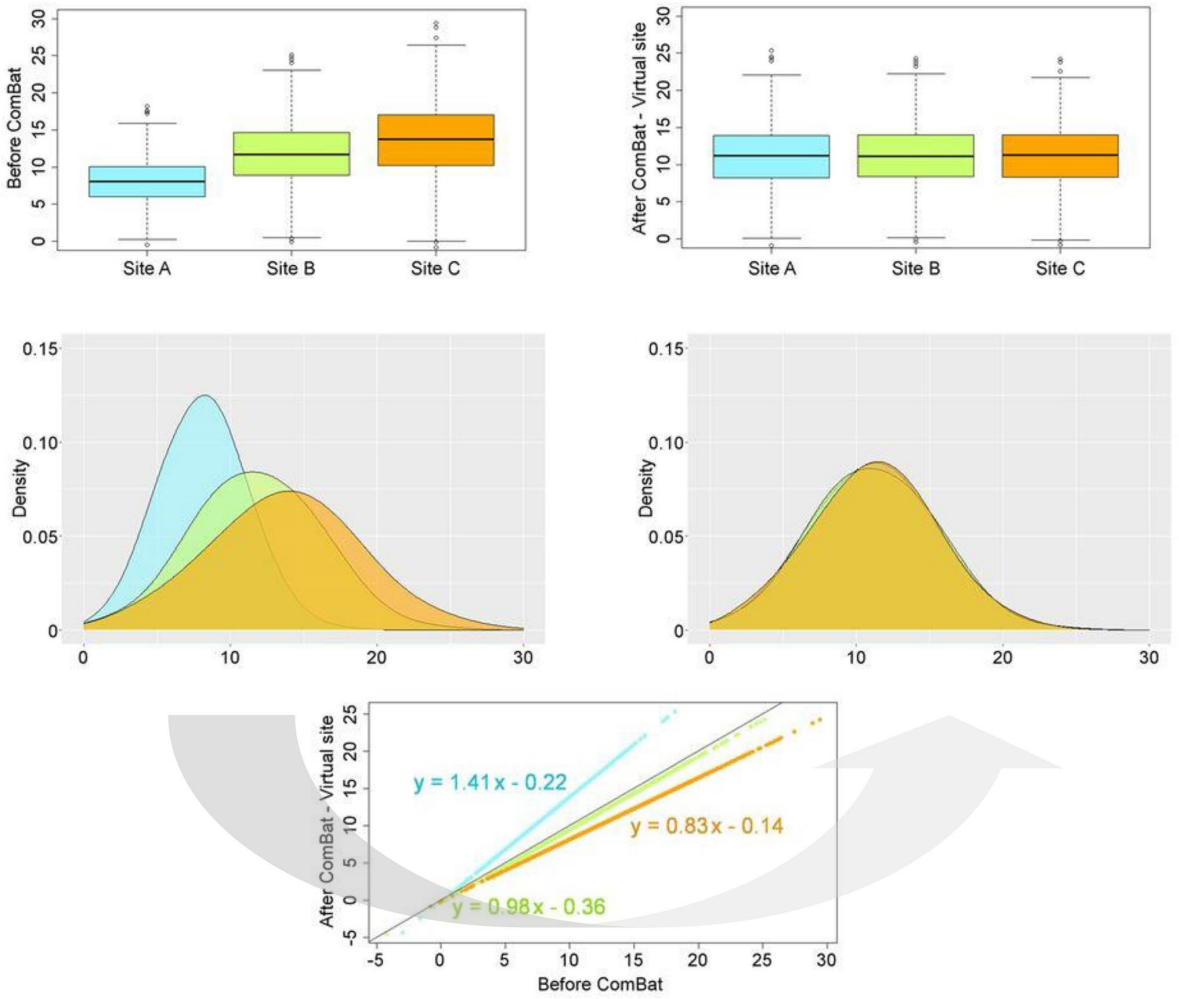
Box plot and quantitative value distributions for data of three virtual sites. After applying the ComBat harmonization method, data on the three sites showed similar distributions. The figures are reprinted with modification from the paper by Orlhac et al. *J Nucl Med*. 2022; 63: 172–179 [74]. © SNMMI

Another representative data-driven approach is the ComBat harmonization method [23], which was originally proposed to reduce “batch effects” in the field of genomics [73]. ComBat directly applies to quantitative values derived from PET images; therefore, additional image data processing and any phantom data acquisition are not mandatory. Orlhac et al. [74] provided a practical guide and list of limitations when applying ComBat to image-derived quantitative metrics. Figure 6 shows simulated data before and after using ComBat. In a multicenter study, Dissaux et al. [75] applied ComBat to numerous FDG-PET/CT radiomic features derived from four different scanners. They reported two radiomic features associated with local control in NSCLC patients undergoing stereotactic body radiation therapy. Hotta et al. [76] also used ComBat to evaluate FDG-PET/CT textural features of primary tumors that were acquired by three different scanners. They evaluated the prognostic value of pretreatment FDG-PET/CT for patients with surgically treated rectal cancer. Gray-level co-occurrence matrix entropy, which presents intra-tumoral metabolic heterogeneity, was associated with overall survival and progression-free survival.

### PET/MR harmonization initiatives

Hybrid PET/MR systems can provide superior soft tissue contrast by MR and functional information by PET at the same position. Such combined information is useful in various fields including neurology, neuro-oncology, cardiology, and oncology [77–79]. The first commercial clinical PET/MR was the Ingenuity TF PET/MR (Philips), a separated-type system [80]. The Ingenuity TF PET/MR has a sequential configuration that connects two subsystems spatially separated by a shared patient bed, so simultaneous PET and MR imaging is not possible. Subsequent integrated PET/MR systems such as the Biograph mMR (Siemens), SIGNA PET/MR (GE), and uPMR790 (United Imaging) allow simultaneous imaging [81–83].

One of the key challenges of PET/MR imaging is MR-based attenuation correction (MR-AC). A segmentation-based method has been proposed to provide a four-compartment body segmentation including air, lung, fat, and soft tissue using the Dixon MR-sequence [84]. However, the segmentation-based methods do not account for bone structures, because a near-zero signal is obtained from bone regions due to bone having both a low spin density and a rapid T2 relaxation rate [85, 86]. Inaccurate bone consideration leads to large underestimation of PET uptakes, especially in near-bone tissues [87]. To overcome this problem, ultrashort echo-time (UTE) and zero echo-time (ZTE) MR-sequences have been developed to capture bone information, and these sequences provide more accurate attenuation maps [88, 89]. More recently, deep learning-based techniques are offering the option to improve accuracy of attenuation correction without CT images [90–92].

**Fig. 7:**
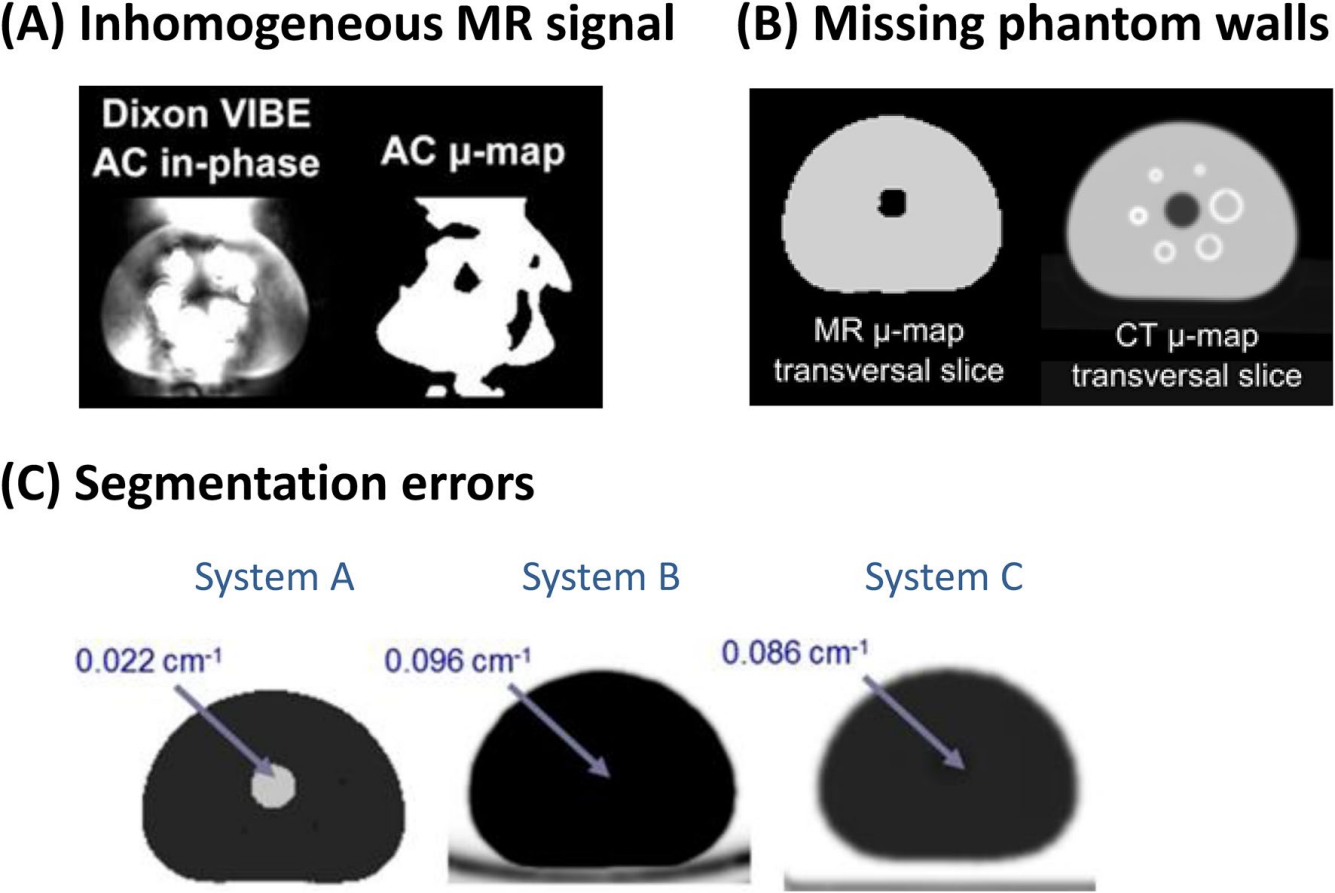
Overview of MR-AC problems: inhomogeneous MR signal (**A**), missing phantom walls (**B**), and segmentation errors (**C**). The images are reprinted with a modification from the following papers: Ziegler et al. [93]. © SNMMI (A), Boellaard et al. [94]. © Am. Assoc. Phys. Med. (B), and Ziegler et al. [95]. Copyright © 2015, Ziegler et al. CC BY 4.0

Although phantom measurements are necessary for scanner quality control and harmonization of quantitative metrics, there are no standard phantoms for PET/MR hybrid imaging. Figure 7 overviews MR-AC problems in phantom measurements. Ziegler et al. [93] reported that a strong artifact was caused by an inhomogeneous radiofrequency excitation MR signal due to a large amount of water as the phantom fluid. Errors in segmentation result in incorrect recognition of water, fat, and air. Boellaard et al. [94] noted that the lung insert was missing in some MR-AC maps. Then, it was found that phantom walls could not be visualized in standard MR-sequences, because they were made of plastic or glass materials [95]. These issues should be solved for valid phantom assessment. Although adding NaCl and NiSO_4_ solutions may improve the homogeneity of MR signals [93], it is still difficult to solve the problem of the missing phantom wall. Ziegler et al. [95] suggested that CT-based AC was suitable for accurate PET performance measurements of PET/MR systems when using the NEMA NU-2 image quality phantom.

Even though MR-AC accuracy was not verified, Laforest et al. [96] harmonized contrast recovery coefficients (CRCs) between the SIGNA PET/MR and Biograph mMR using the NEMA NU-2 image quality phantom. Six customized spheres (diameters: 8.5, 11.5, 15, 25, 32.5, and 44 mm) were used in addition to the standard six spheres (diameters: 10, 13, 17, 22, 28, and 37 mm). The CT-based AC methods were used and CRCs were evaluated together with the root-mean squared discrepancy (RMSD) to determine harmonized reconstruction parameters. Laforest et al. achieved high CRCs within the limits of EARL1 and minimized the variation between two different PET/MR scanners. Jentzen et al. [97] evaluated quantitative accuracy of ^124^I PET/MR images for patients with differentiated thyroid cancer using serial PET/CT data as a reference. After harmonization processing, activity concentrations of lesions in the neck were comparable between images measured with PET/CT (CT-AC) and PET/MR (MR-AC).

For evaluation of brain PET imaging, the Hoffman 3D brain phantom has been widely used as mentioned above. Since the phantom is composed of acrylic plates, MR-AC accuracy cannot be evaluated as well as for the NEMA NU-2 image quality phantom. Ribeiro et al. [98] evaluated PET image quality of PET/MR systems using the Hoffman 3D brain phantom with CT-based template AC. On the other hand, Teuho et al. [99] used another 3D brain phantom (the Iida phantom [55]) which contains the gray matter, white matter, skull, and tracheal structures with a realistic head contour. Teuho et al. evaluated the difference between CT-AC and MR-AC on visual and quantitative differences using multiple PET/MR and PET/CT systems. They noted that regional differences between PET/MR and PET/CT systems were minimized using CT-AC. Because white matter of the 3D brain phantom is composed of polymer, which is not visible in MR-AC, the Iida phantom is still insufficient for evaluation of MR-AC accuracy [100].

CT-AC is the current standard method in PET/MR phantom studies for evaluating image quality and quantitative accuracy, thereby aiming at harmonization. In human PET/MR studies, however, MR-AC is generally used to reconstruct PET images. A PET/MR multimodal phantom, which mimics the electron density and MR contrast of human tissue, is required to evaluate MR-AC accuracy, and facilitate PET/MR harmonization [100]. Harries et al. [101] developed a realistic phantom of the human head using water-saturated gypsum plaster, silicone, agarose gel, etc. Rausch et al. [102] developed a cylindrical phantom with a 3D printable MR-visible polymer. Preliminary evaluations of MR-AC accuracy may be possible using these phantoms although they still cannot provide PET signals. On the other hand, Canata et al. [103] proposed to use a patient as a phantom to assess the MR-AC accuracy using CT as the reference. PET/MR harmonization will be facilitated if a standard method that can verify the MR-AC accuracy is established.

### Non-^***18***^F PET harmonization initiatives

Many novel radioisotopes have emerged for diagnostic PET imaging, theranostics, and immuno-PET imaging. One of these radioisotopes for PET is ^68^Ga. ^68^Ga-labelled tracers targeting somatostatin receptor, prostate-specific membrane antigen (PSMA), and fibroblast activation protein inhibitor (FAPI) have been used in recent clinical studies [104–106]. These tracers are often used in combination with ^177^Lu and ^225^Ac as part of theranostics [105, 107, 108]. ^124^I has been used to evaluate lesion dosimetry prior to radioiodine treatment in patients with differentiated thyroid cancer [97, 109]. Immuno-PET is a promising tool for predicting the outcome of monoclonal antibody-based cancer therapy using such radioisotopes as ^64^Cu, ^74^Br, ^86^Y, ^89^Zr, and ^124^I [110, 111].

It is essential to consider the physical properties of each radioisotope for harmonization of non-^18^F PET imaging. Table 3 lists the physical properties of typical positron emitting isotopes [112–115]. Long positron ranges due to the high energy of emitted positrons lead to blurring of the source distribution and loss of spatial resolution [116, 117]. A low positron branching ratio may result in inferior image quality and errors in quantitative uptake measurements due to the low count statistics and the cross-calibration error. In addition, some radioisotopes emit prompt or non-prompt γ rays in the decay process (Table 3). If such associated γ rays have contaminated coincidence count data in energy and coincidence timing windows, they will lead to degradation of image quality and quantitative accuracy [113].

**Table 3:** Properties of typical positron emitting radioisotopes

Radioisotopes	Half-life	β + branching ratio	β + max energy	β + mean range (in water)	Γ branching ratio	Γ mean energy
^18^F	109.8 min	96.7%	0.634 MeV	0.6 mm	–	–
^11^C	20.4 min	99.8%	0.960 MeV	1.2 mm	–	–
^64^Cu	12.7 h	17.5%	0.653 MeV	0.7 mm	–	–
^68^Ga	67.8 min	88.0%, 1.2%	1.899, 0.821 MeV	3.1 mm	3.2%	1.08 MeV
^89^Zr	78.4 h	22.7%	0.902 MeV	1.3 mm	100%**	0.909 MeV**
^124^I	100.2 h	11.7%, 10.7%	1.535, 2.138 MeV	3.0 mm	62.9%, 11.2%	0.603, 1.70 MeV
^90^Y	64.1 h	0.0032%*	0.769 MeV	N/A	–	–

*Branching ratio of the e + /e− pair production, **Not prompt *γ*-rays

**Fig. 8:**
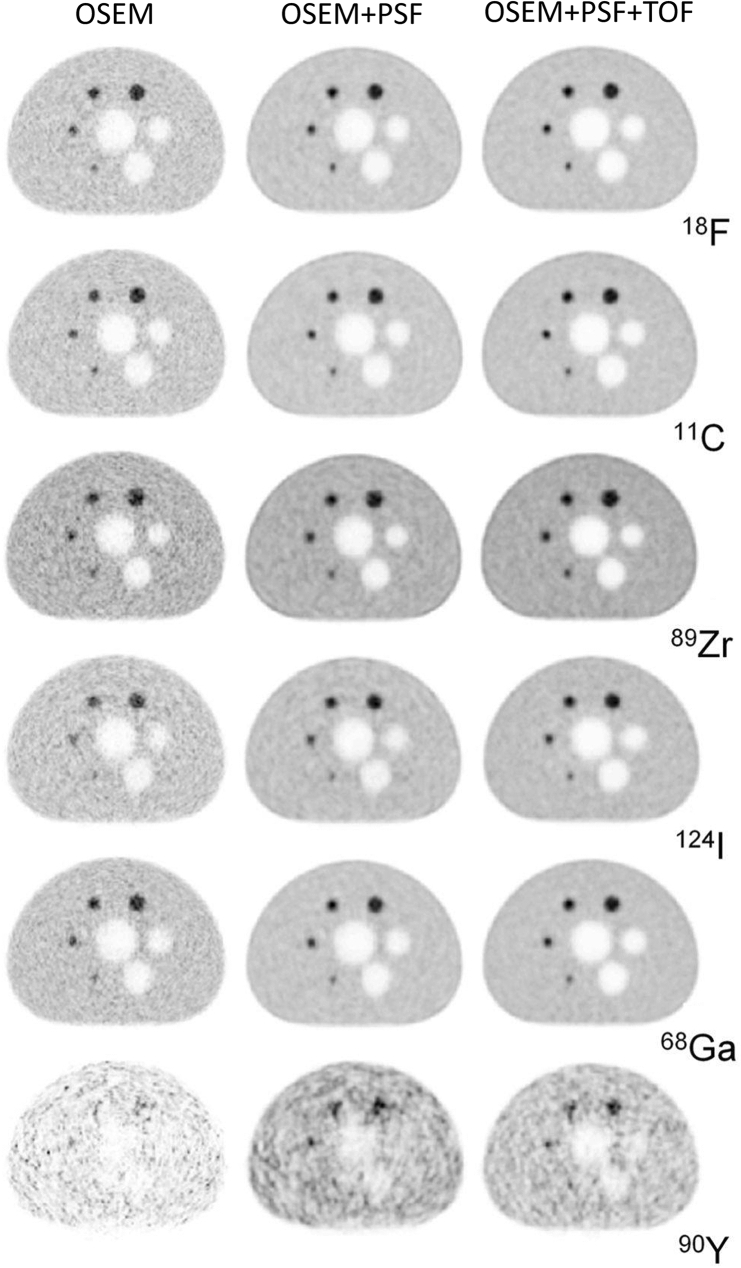
PET images of the NEMA NU-2 image quality phantom filled with various radioisotopes. The images were acquired by Biograph mCT (Siemens). The images are reprinted with modification from the paper by Soderlund et al. [113]. © SNMMI

Some studies have investigated PET image characteristics of different radioisotopes [114, 118]. Soderlund et al. [113] evaluated the image quality and spatial resolution for a set of radioisotopes (^18^F, ^11^C, ^89^Zr, ^124^I, ^68^Ga and ^90^Y). Figure 8 shows PET images of the NEMA NU-2 image quality phantom filled with the various radioisotopes. ^124^I and ^68^Ga, which have longer positron ranges, showed slight degradation of contrast recovery and resolution in small spheres compared to ^18^F. Other reports using small animal PET systems showed considerable image blurring due to the positron range [119, 120]. Especially for ^124^I, the image quality may be deteriorated due to prompt γ-rays having the energy (603 keV) that is similar to 511 keV of annihilation photons.

Harmonization studies on non-^18^F PET imaging have been conducted by several groups. Huizing et al. [121] performed ^18^F and ^68^Ga PET phantom acquisitions using 13 PET/CT systems and evaluated quantitative recovery coefficients according to the EARL strategy. While ^18^F recovery coefficient curves for all PET/CT systems satisfied the range for EARL1 standards, ^68^Ga curves were located near the lower limit of the range. After correcting the difference between ^68^Ga and ^18^F cross-calibrations, ^68^Ga recovery coefficient curves for most scanners satisfied the EARL1 standards. Some investigators worked on multicenter ^89^Zr PET harmonization studies. Makris et al. [122] and Kaalep et al. [123] used the NEMA NU-2 image quality phantom with a 10:1 sphere-to-background ratio according to the EARL protocol. Results from both studies showed that inaccuracy of the local dose calibrator cross-calibration would be a large source of bias in quantitative values. Christian et al. [124] used an anthropomorphic chest oncology phantom filled with ^89^Zr at a clinically relevant activity level and a 4:1 sphere-to-background ratio. They investigated optimal reconstruction parameters based on visual lesion detectability and SUVpeak recovery coefficients.

Minimizing measurement errors and cross-calibration errors is essential for harmonization of non-^18^F PET imaging. In most PET/CT systems, cross-calibration between the dose calibrator and a PET system is performed using ^18^F, which may result in inaccurate quantification with radioisotopes other than ^18^F. Accurate quantification for non-^18^F radioisotopes requires proper correction of such physical properties as branching ratio, half-life, and prompt γ rays. Bailey et al. [125] and Sanderson et al. [126] noted most PET systems underestimated ^68^Ga SUVs which was caused by overestimation of ^68^Ga radioactivity using dose calibrators with a default calibration factor setting. Appropriate ^68^Ga calibration factor setting is important to provide accurate quantitative values. Beattie et al. [127] recommended the use of appropriate dose calibration factors for measuring ^89^Zr and ^124^I. For ^124^I, they proposed to use a copper filter to remove the contribution of X-rays emitted by ^124^I in the 20–40 keV range.

As mentioned earlier, additional care is required for harmonization of non-^18^F PET. Appropriate quality control for PET systems, dose calibrators, and other associated devices should be performed considering their physical properties. Ideally, scanner validation and harmonization are conducted with the radioisotopes being used in the clinical protocol [124].

### Future challenges and summary

**Fig. 9:**
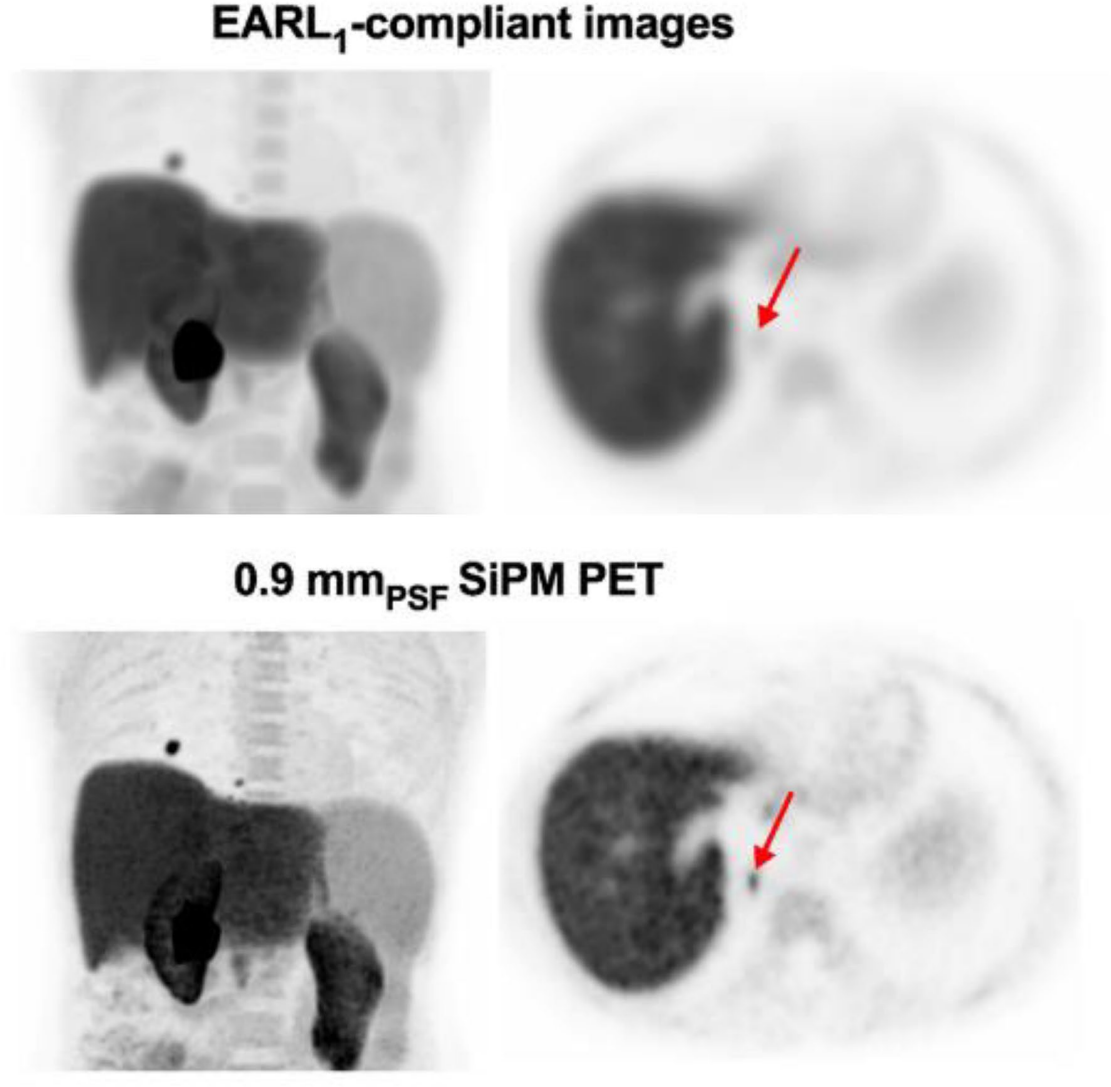
^18^F-fluorocholine PET images acquired by Biograph Vision scanner (Siemens). The patient was referred for restaging after a liver graft for hepatocellular carcinoma. PET data were reconstructed with an EARL-compliant harmonized parameter (9 mm Gaussian filter) (upper) and with point-spread function modeling and 0.9 mm voxel size (lower). The red arrow indicates a tiny lung metastasis. The images are reprinted with modification from the paper by Aide et al. [128]. Copyright © 2021, Aide et al. CC BY 4.0

The major harmonization approach is adjusting image reconstruction parameters and smoothing filters so that quantitative values are comparable among different scanners. It should be noted that such harmonization methods may spoil small lesion detectability. Aide et al. [128] presented a representative case before and after harmonization, which used a state-of-the-art SiPM-based PET/CT scanner for measurements (Fig. 9). To mitigate deteriorating small lesion detectability, harmonization criteria should be regularly updated in step with scanner performance improvements [11]. Because novel PET systems such as SiPM-based scanners [129, 130], total-body scanners [131, 132], and brain-dedicated systems [133, 134] have been increasingly applied to clinical studies, harmonization methods should adapt to such imaging systems.

Many organizations have built harmonization strategies; however, international methodology harmonization is necessary to achieve harmonization of quantitative PET, because clinical studies and trials are conducted worldwide. International harmonization of the standards remains as an issue to ensure the comparability of quantitative values in PET.

This review discussed harmonization strategies for quantitative PET. To make use of PET as a biomarker, quantitative values derived from PET should be comparable between scanners, sites, and studies. By minimizing the bias and variability due to technical, biological, and physical issues, quantitative PET can precisely highlight physiological and pathological changes even with small datasets. It is expected that this review will facilitate harmonization of quantitative PET and PET-derived biomarkers contribute to research and development in medicine.

## Data Availability

Data presented in this review are available upon reasonable request.
